# Super-Resolution Imaging Conditions for enhanced Yellow Fluorescent Protein (eYFP) Demonstrated on DNA Origami Nanorulers

**DOI:** 10.1038/srep14075

**Published:** 2015-09-16

**Authors:** Ija Jusuk, Carolin Vietz, Mario Raab, Thorben Dammeyer, Philip Tinnefeld

**Affiliations:** 1 Institute for Physical & Theoretical Chemistry, and Braunschweig Integrated Centre of Systems Biology (BRICS), and Laboratory for Emerging Nanometrology (LENA), Braunschweig University of Technology, Braunschweig 38106, Germany

## Abstract

Photostability is one of the crucial properties of a fluorophore which strongly influences the quality of single molecule-based super-resolution imaging. Enhanced yellow fluorescent protein (eYFP) is one of the most widely used versions of fluorescent proteins in modern cell biology exhibiting fast intrinsic blinking and reversible photoactivation by UV light. Here, we developed an assay for studying photostabilization of single eYFP molecules with respect to fast blinking and demonstrated a 6-fold enhanced photostability of single eYFP molecules with a beneficial influence on the blinking kinetics under oxygen removal and addition of aliphatic thiols (dSTORM-buffer). Conjugation to single stranded DNA and immobilization via DNA hybridization on a DNA origami 12 helix bundle in aqueous solution allowed photophyiscal studies of eYFP at the single-molecule level and at close to physiological conditions. The benefit of improved photophysical properties for localization-based super-resolution microscopy is demonstrated and quantitatively characterized by imaging 12 helix bundle DNA origami nanorulers with binding sites at designed distances of 160 and 100 nm and by imaging microtubules in fixed mammalian Vero cells.

Fluorescent proteins have become the most important labels that drive innovation in cell biology^1^,^2^,^3^. Besides the expanding color pallet, more functions are engineered into different derivatives of fluorescent proteins making them robust, quickly maturating, monomeric, switchable or photoactivatable^2^. The latter properties have especially been important for recent breakthroughs in super-resolution microscopy that overcomes the diffraction limit by localizing all molecules in a specimen successively^4^. Interestingly, also some of the classical early fluorescent proteins such as eYFP could be switched under certain conditions and even at the single-molecule level^5^. While photoswitching mechanisms and intensity fluctuations could be ascribed to cis-trans isomerization or deprotonation^3^,^6^, much less is known about how the environmental conditions influence the photophysical properties of fluorescent proteins^7^,^8^,^9^. eYFP is especially useful for super-resolution microscopy because it is one of the brightest fluorescent proteins and it is widespread alleviating the need of creating new protein fusions. Many different fluorescent and non-fluorescent states have been reported for eYFP that have been assigned to e.g. a protonation/deprotonation equilibrium^5^,^10^,^11^,^12^,^13^. A longer lived OFF-state could be activated with UV illumination. Both the fast blinking and the UV-activatable blinking have been used for super-resolution microscopy^13^,^14^. However, use of UV-reactivation pulses might enhance the photodamage of biological samples, impair the measurement time and unnecessarily complicate the measurement process due to alternation of laser excitation.

Here, we investigated the influence of buffer compositions on the photostability of single eYFP molecules with a special focus on the compatibility with imaging conditions used in super-resolution microscopy for organic dyes^15^,^16^,^17^. For these purposes we modified eYFP molecules with DNA linkers and immobilized them on DNA origami nanostructures in solution. We identified a strong buffer dependence of the eYFP-photostability and the optimal condition interestingly coincides with those used for the established super-resolution technique dSTORM^17^. Based on these findings we constructed the first fluorescent protein DNA origami nanorulers^18^,^19^,^20^ with intermark separations of 100–160 nm and imaged them using fast eYFP blinking under optimized conditions. DNA origami nanorulers equipped with organic dyes have already become a useful tool as reference structures for super-resolution microscopy methods^18^,^19^,^21^. Their reproducible structural homogeneity, chemical robustness, suitability as biocompatible surfaces^22^ and controllable labeling density enable careful quantitative characterization and comparison of different fluorophores or imaging conditions.

## Results

### Photostability study of single eYFP

**Figure 1:**
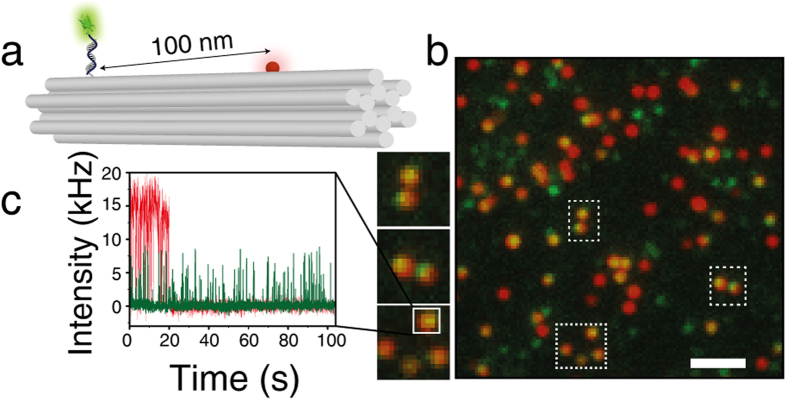
(**a**) Sketch of the 12 helix bundle DNA origami (220 × 14 × 14 nm^3^) equipped with a red ATTO647N dye and a fluorescent protein attached by hybridizing a DNA modified eYFP to a single stranded protrusion. (**b**) Corresponding TIRF image of overlaid channels of eYFP-emission (green) and ATTO647N-emission (red). Colocalized dyes appear yellow in the false-color image, scale bar 2 μm. (**c**) Representative single-molecule fluorescence transient colocalized eYFP and ATTO647N measured in dSTORM-buffer.

In order to reduce the influence of the environment on the fluorophore (e.g. embedded in polymers), we immobilized the fluorescent proteins in aqueous solution which has rarely been done in previous studies of single fluorescent proteins^23^. To this end, we first engineered an eYFP variant^24^ with a cysteine at the C-terminal end and covalently coupled the eYFP to a single stranded DNA via a heterobifunctional crosslinker (sSMCC, see Supplementary Information for sample preparation)^25^. To further reduce the influence of the surface on the properties of the fluorescent proteins we hybridized the DNA-eYFP conjugates to complementary DNA-strands protruding from a 12 helix bundle (12HB) DNA origami nanostructure (see sketch in Fig. 1a)^19^. DNA origamis have previously been proven to be suitable biocompatible surfaces^22^. The 12 helix bundle DNA origami^26^ is also easily modified with another spectrally distinct fluorescent dye (here ATTO647N). Colocalization of the eYFP fluorescence with the ATTO647N allows unequivocal discrimination of single eYFP proteins against an unavoidable fraction of impurity molecules with similar brightness. This was necessary as single fluorescent proteins commonly are not brighter than typical impurity molecules in this wavelength range^27^. The 12HBs additionally contained 4 biotin-modified DNA-strands for immobilization on BSA-biotin/NeutrAvidin coated cover slips^19^.

**Figure 2:**
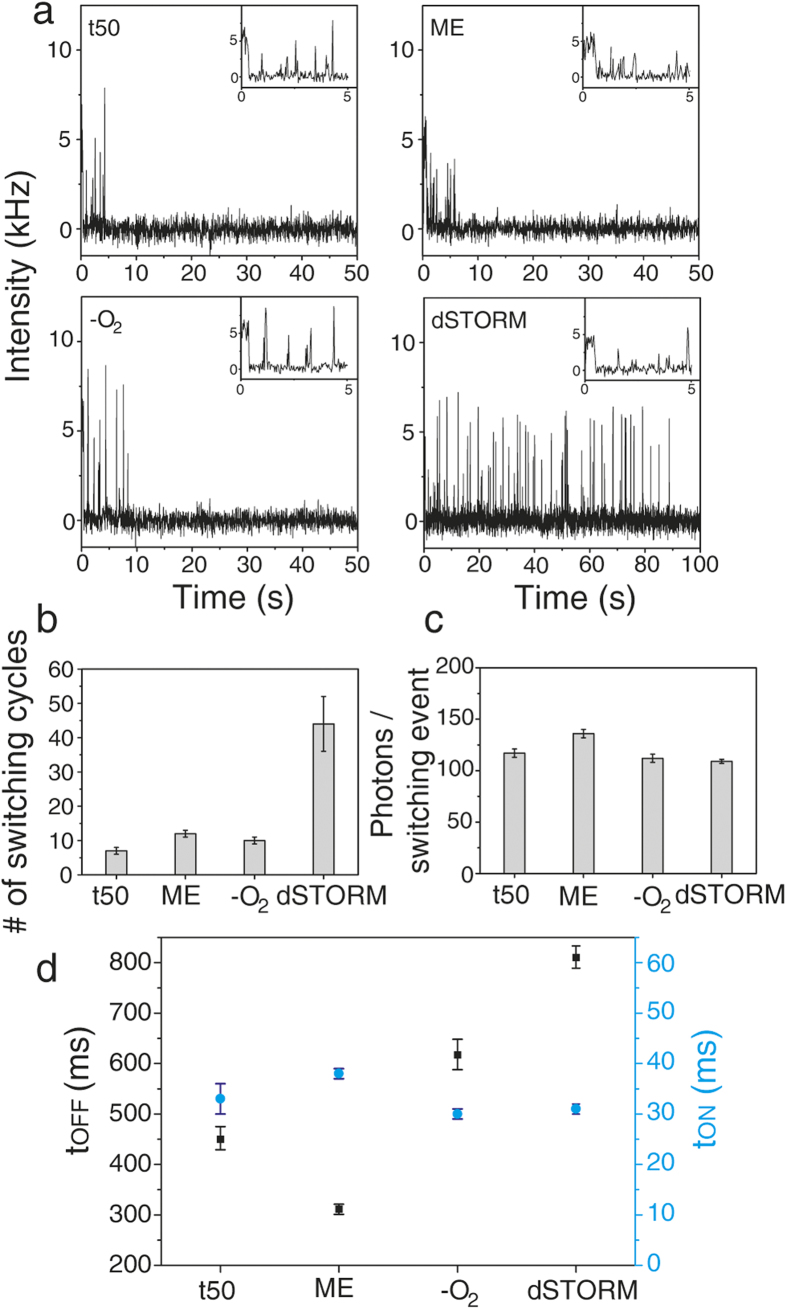
(**a**) Representative fluorescence transients of eYFP in four different buffer conditions: t50 (Tris, pH 7.4), 70 mM ME, enzymatic depletion of oxygen, and dSTORM-buffer (depletion of oxygen and 70 mM ME). (**b**) Mean number of switching cycles of single eYFP prior to bleaching. (**c**) Mean number of photons of eYFP per ON-time. (**d**) Mean lifetimes of ON- and OFF-states, t_ON_, t_OFF_ of single eYFPs. (**b**–**d**) Mean values were obtained from exponential fitting of histograms of more than 100 individual proteins colocalized with one ATTO647N dye. The error of the fits is indicated.

Figure 1b shows the two-color fluorescent image of eYFP modified 12HBs with eYFP-emission (green) and ATTO647N-emission (red) recorded with a custom-built objective-type total internal reflection fluorescence microscope with 488 nm, (ca. 800 Wcm^−2^) and 644 nm (ca. 300 Wcm^−2^) excitation and 405 nm (ca. 500 Wcm^−2^) as optional reactivation source with 30 ms time resolution. In order to reduce drift we used an actively stabilized table and a nosepiece to mechanically couple objective and specimen^28^. Yellow spots indicate colocalization of eYFP and ATTO647N on the same DNA origami. These spots were considered for analysis. A representative fluorescence transient is depicted in Fig. 1c, which shows ATTO647N bleaching after ~19 s and eYFP blinking over a time period of more than 100 s. Starting from t50-buffer (50 mM Tris, 50 mM NaCl, 100 mM MgCl_2_, pH 7.4), we studied the influence of oxygen removal, thiols (β-mercaptoethanol, ME) and a combination of oxygen removal and ME, which has become known as dSTORM-buffer^17^. As expected, eYFP exhibited an intrinsic blinking behavior on the time scale of milliseconds (Fig. 2a). Interestingly, a closer look reveals that eYFP exhibits an initial longer ON-state (~0.5 s) under all four buffer conditions (Fig. 2a, insets). Obviously, the quickly blinking state is first formed from the original eYFP in an irreversible photoreaction. In t50-buffer eYFP entered a reversible or irreversible dark state typically within 5 s. Addition of 70 mM ME or enzymatic depletion of oxygen by glucose oxidase and catalase (GOC) did not lead to a notable change of photostability or blinking properties. In dSTORM-buffer (oxygen depletion by GOC, 70 mM ME), however, eYFP exhibited an enhanced photostability and bleached after much longer exposure time of ca. 100 s (Figs 2a and 1c). This photostabilizing effect can be visualized in terms of number of switching cycles which eYFP undergoes before photobleaching (Fig. 2b) or by the overall number of detected photons (see Figure S3). Compared to t50-buffer a 6-fold increased photostability was observed with dSTORM-buffer. Besides the enhanced photostability, the photophysical characteristics of eYFP remained nearly unchanged. Blinking started after an initial longer ON-state, the number of photons per switching event (ca. 120 photons, Fig. 2c) and the average lifetime of the ON-state remained constant (30 ms, Fig. 2d, the initial long ON-states were excluded from analysis). However, the average lifetime of the OFF-state increased in dSTORM-buffer. With respect to super-resolution microscopy, increased photostability and lower duty cycle are some of the most important properties of fluorescent probes and determine the quality of super-resolved images in terms of high localization density^29^,^30^,^31^,^32^.

### Super-resolution with eYFP on DNA origami

**Figure 3:**
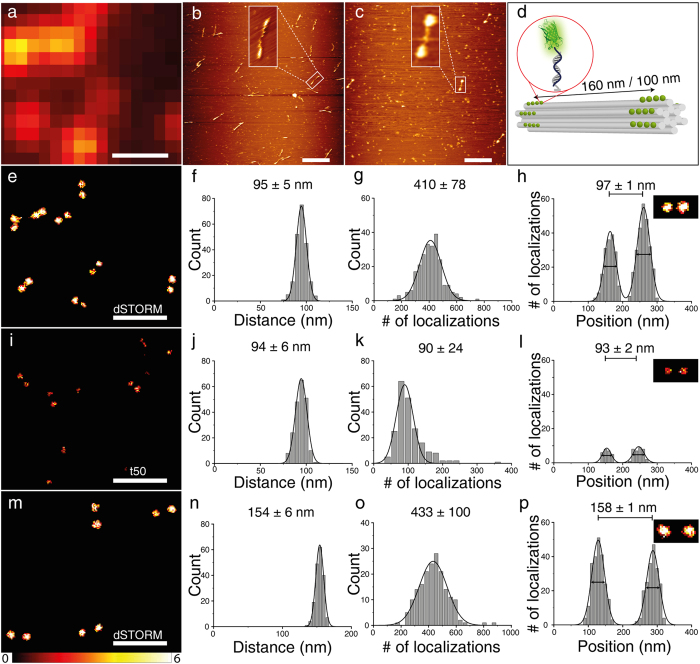
(**a**) TIRF-image of 12 helix bundle DNA origamis with eYFP-DNA conjugates with a designed distance of 100 nm, scale bar 500 nm which corresponds to the super-resolved image in 3e. (**b**) AFM image of 12HB DNA origamis (160 nm). (**c**) AFM image of 12HB DNA origamis with eYFP-DNA conjugates (160 nm). Background spots are due to BSA used to increase filtering yields, scale bar 500 nm. (**d**) Sketch of 12HB DNA origamis with designed distances of 100 nm or 160 nm between two marks whereas each mark is created by 16–19 single stranded protrusions. Super-resolution imaging of eYFP labeled 12HBs (100 nm and 160** **nm) was obtained by successive localization of blinking molecules (**e**–**p**)^31^. (**e**,**i**,**m**) Super-resolution images. (**f**,**j**,**n**) Histograms of intermark distances. (**g**,**k**,**o**) Overall number of localizations per 12HB DNA origami. (**h**,**l**,**p**) Cross sections of pixels indicated in inset in dSTORM-buffer (FWHM 39/42 nm in (**h**), 42/42 nm in (**p**)) and in t50-buffer (FWHM 31/38 nm in (**l**)), respectively. More than 200 structures were analyzed for each distance and buffer condition. Errors in (**f**–**h**,**j**–**l**,**n**–**p**) are standard deviation of Gaussian fits.

To demonstrate the importance of the improved photophysical properties in dSTORM-buffer, we expanded the eYFP modified 12HB into a nanoruler by creating two marks at distances of 100 or 160 nm. One mark was created by 19 single-stranded protrusions (160/100 nm) and the other mark was created by 16 and 18 docking sides for 100 nm and 160 nm, respectively (Fig. 3d)^19^. DNA origami nanorulers have become established reference structures for assessing the quality of super-resolution microscopy methods^18^,^19^,^33^. AFM images in Fig. 3b,c show the 12HB adsorbed on mica without and with hybridized eYFP-DNA conjugates at an intermark distance of 160 nm. The background spots also visible in the image were attributed to the presence of BSA in the filtering buffer during purification after labeling which was necessary to avoid loss of eYFP-12HB due to the sticking of protein-DNA conjugates to the filter surface. Still, several dumbbell structures representing eYFP labeled 12HB are visible demonstrating successful nanoruler production. From fluorescence intensity and the number of localizations we deduced a labeling efficiency of 25–40% referring to the overall number of protruding single strands per 12HB origami nanostructure. eYFP-12HB nanorulers were characterized by localization-based super-resolution microscopy (Fig. 3e–p). Whereas the nanorulers are hardly recognized for measurements in t50-buffer (Fig. 3i) bright and robust double-spot structures are found for measurements in dSTORM-buffer (Fig. 3e,m). Additionally, there are several single-spots in 3i instead of double-spots which is due to fast photobleaching of eYFP in t50-buffer. Measurements in t50-buffer with 70 mM ME or oxygen depletion separately could not improve significantly the quality of super-resolution images (Supplementary Figure S4). Additionally, reactivation by UV and subsequent excitation with 488 nm of eYFP in t50-buffer increased the localization density only by a factor of ca. 2 (Supplementary Figures S4,S6). The determined distances match the expectations of the intermark distances (Fig. 3f,j,n Supplementary Figure S4) and a typical localization precision of ca. 40 nm was obtained in accordance with a detected photon number of ca. 120 photons/ON-time^23^,^31^ (Fig. 2c, Supplementary Figure S5). The improved duty cycle and photostability in dSTORM-buffer yield clearly more localizations (Fig. 3g,o) and brighter images (Fig. 3e,m).

### Super-resolution cell imaging with eYFP

**Figure 4:**
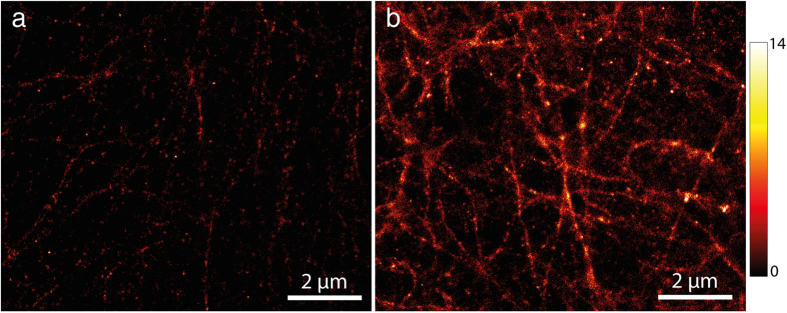
Super-resolved imaging of microtubules fused to eYFP in fixed mammalian Vero cells without (**a**) and with dSTORM-buffer (**b**). Imaging was performed with a commercial objective-type TIRF microscope Leica GSD 3D (excitation at 488 nm, integration time of 30 ms).

In order to verify the reported enhanced photostability of eYFP due to the addition of β-mercaptoethanol and oxygen depletion in cell imaging, we performed analogous super-resolution imaging of microtubules in mammalian Vero cells overexpressing eYFP-tubulin. Figure 4(a,b) shows the images obtained from cells in t50-buffer and in dSTORM-buffer, respectively. Identical microscope settings and analysis parameters were used for both images that were obtained from the same sample. Obviously, the increased number of localizations and the increased fraction of eYFPs contributing to the reconstructed image reveal more cellular structural information when imaged in dSTORM-buffer.

## Discussion

Our data show that the performance of eYFP can be improved by adapting the buffer conditions. We identified dSTORM-buffer (oxygen removal with addition of thiols) as a superior environment for eYFP photostability and blinking. Our findings indicate that dSTORM-buffer prohibits eYFP from entering the long-lived reversible or irreversible dark states resulting in extended fluorescence transients and higher localization density. Since the reactivation by UV in Tris-buffer did not lead to a significantly improved performance of eYFP in the super-resolution experiments, we consider UV-associated photodamage of the probe as a reason for this. The higher number of localizations can be used for improved resolution by increasing the detection threshold for single-molecule localization events. If, for example, the single-molecule detection threshold is adapted so that a similar number of localizations (Fig. 3i,k) is obtained for the data shown in Fig. 3e, the resolution in Fig. 3e improves from 42 ± 6 nm to 24 ± 6 nm in dSTORM-buffer. Thus, the achievable resolution can be higher although the average number of photons per localization is not improved. Additionally, dSTORM-imaging buffer enables further significant advantages with respect to e.g. simplicity of the imaging conditions. Alternation of excitation and reactivation wavelengths becomes obsolete. For this work relatively short imaging times of ~4 min were sufficient to obtain super-resolved images with a high localization density. The results from our photophysical studies were directly reflected in the super-resolution images of the first fluorescent protein nanorulers. An additional advantage of this method is the possibility to perform multi-color-dSTORM imaging in combination with organic dyes. As proof-of-principle we successfully carried out 2-color-dSTORM-imaging on 12HB DNA origamis with eYFP and Alexa 647 (Figure S7). The eYFP-DNA conjugates have enabled extending the DNA origami nanoruler family by the important class of fluorescent proteins. Additionally, we show the applicability and reproducibility of the reported stabilizing effect in fixed cells.

## Methods

### Sample preparation

For engineering of enhanced eYFP expression units we recruited the established eYFP sequence (pEYFP-C1 Vector, Clonetech, Takara) containing GFP-10C mutations^34^,^35^ and introduced the A207K dimer interface breaking mutation^27^,^36^. The reverse back-translated amino acid sequence for optimized translation in *Escherichia coli* was used as synthetic DNA sequence^24^. Expression plasmid pTDeYFP-His_6_-Cys was constructed by PCR-amplification of the synthetic sequence with oligonucleotides FW-GGA **C**CATGG TT TCTAAAGGTGAAGAACTGTTCACCGGTGTTGTTCCG and rev-GTC AAGCTT TCA GCA GTGGTGGTGGTGGTGGTG TTTGTACAGTTCGTCCATACCCAGGGTGATACCAGCAGC to attach the labeling accessible cysteine residue, His_6_-tag and restriction endonuclease cleavage sites. Phusion™ DNA Polymerase (Thermo Scientific) was used for amplification and products were cloned into pET28a(+) (Novagen) *via Nco*I and *Hind*III sites (underlined).

The plasmid pTDeYFP-His_6_-Cys was heat shock transformed in *E.coli* BL21 cells. From overnight precultures, expression cultures were inoculated 1:100 in LB medium selecting with kanamycin 50 μg/ml and cultured at 37 °C until they reach OD600 of 0.3. Prior to induction we chill the cultures to 22–25 °C and induce at OD600 of 0.5 with 125 μM isopropyl-β-D-thiogalactopyranoside (IPTG). Cells are harvested by centrifugation after overnight expression, lysed using bacterial protein extraction reagent (Perbio Science) and initially purified *via* immobilized metal affinity chromatography on Ni-NTA agarose (Qiagen).

The hetero-bispecific crosslinker sulfosuccinimidy 4-[N-maleimidomethyl]-cyclohexane-1-carboxylate (sSMCC) was used to conjugate the protein to aminomodified DNA oligonucleotide (5′-NH2-C6H12-GTG ATG TAG GTG GTA GAG GAA) as previously suggested (Supplementary Figure S2)^25^.

12HB DNA origami nanostructures were produced by mixing p8064 scaffold (10 nM), unmodified DNA staple strands (10x molar excess) and modified DNA staple strands (30x molar excess) in folding buffer (5 mM Tris, 1 mM EDTA, 16 mM MgCl_2_, pH 8) with a total volume of 100 μL. All DNA sequences are listed in Supplementary Information. The folding reactions was carried out in 200 μl PCR tubes in a Thermal Cycler (MJ Research, PTC-225) and the following program: 1) 80 °C, 15 min, 2) 80 °C - 66 °C, 5 min per °C, 3) 65 - 30 °C, 30 min per °C^19^. Excessive staple strands were removed using centrifugal filters (Amicon, UFC510096, 100 k) by filtering and washing the folding solution three times with folding buffer (0.5 mL; 10 min, 10000 rcf). In the final step, the 12HB origamis were recovered by centrifugation of the flipped tube at 3000 rcf for 15 min. For labeling with eYFP-DNA-conjugate, ca. 5 fold excess per protruding staple and 50 μL folding buffer were added to the filtered 12HB DNA origamis (30 μL, ca. 10 nM) and incubated for 2 h at 37 °C. Before purification of labeled 12HB origamis an Amicon filter was washed with folding buffer containing 0.1 mg/mL BSA in order to reduce sticking of eYFP-12HB origamis to the filter material. eYFP-12HB origamis were purified by filtering and washing three times with folding buffer containing 0.1  mg/mL BSA (10 min, 8000 rcf). Integrity of 12HB origamis was verified by atomic force microscopy before and after labeling with eYFP-DNA (Fig. 3b,c). For 2-color super-resolution imaging the labeling procedure was extended by an additional incubation with 5′-Alexa647-AAAAAAAAAAAAAAAAAAAAAA and removal of unbound staples by filtering as mentioned above. For TIRF-measurements, we used Nunc Lab-Tek chambered coverglass (Thermo Scientific 155409, # 1.5). Lab-Tek chambers were incubated with 1 M KOH for 5 min, washed three times with PBS buffer, incubated with 100 μL BSA-biotin (0.5 mg/mL in PBS) over night at 4 °C, washed again three times with PBS buffer and incubated with NeutrAvidin (0.5 mg/mL in PBS) for 1 h at room temperature, washed again with PBS buffer and finally with t50 buffer three times. Purified eYFP-12HB samples were diluted (ca. 1:2) with t50-buffer (final concentration of MgCl_2_ was 100 mM) and incubated for 10 min in Lab-Tek chamber. After this step the Lab-Tek chamber was washed three times with t50-buffer. The oxygen scavenging system (glucose oxidase and catalase, GOC) contains 1 mg/ml glucose oxidase, 0.4% (v/v) catalase, 30% glycerol, 12.5 mM KCl in 50 mM Tris, pH 7.5. For TIRF-measurements, GOC-stock solution was dissolved in t50-buffer, 1% glucose (w/w) to a concentration of 10% (v/v).

### Cell culture and transfection for cell imaging

Mammalian Vero cells were cultured in RPMI medium (Gibco) with 10% FCS (Gibco) at 37 °C and 10% CO_2_. The cells were seeded into LabTeks (Nunc) 24 hours before transfection and transfected with plasmid eYFP-tubulin (human α-tubulin) using FuGENE HD transfection reagent (Promega) according to the manufacturer’s instructions. The eYFP is based on the same sequence (clonetech) used for the eYFP-DNA conjugates but devoid of the A207K dimer interface breaking mutation and was cloned as described for plasmid Citrine-Tubulin in plasmid pEGFP-Tubulin (clonetech)^37^. The cells were fixed and permeabilized using ice-cold methanol for 10 minutes at −20 °C. Cell imaging was carried out ca. 24–48 hours after transfection under different buffer conditions.

### Setup characterization

For all single-molecule measurements (except the cell imaging) presented in this work, we used a custom-built objective-type total internal reflection fluorescence microscope (Olympus IX71 with 1.6 × optical magnification). For fluorophore excitation we used a 644 nm diode laser (iBeam smart/Toptica photonics, clean-up filter Brightline HC 650/13, Semrock), a 488 nm diode laser (iBeam smart/Toptica photonics, clean-up filter 488/1.9, AHF) which were coupled into the microscope by a dichroic beam splitter (zt 647 rdc, Chroma) and (Di01-R488, Semrock), respectively. For two-color super-resolution imaging these two laser lines were coupled by a quad-edge beam splitter (zt405/488/561/640rpc, AHF). For eYFP-reactivation, a 405 nm diode laser (iBeam smart/Toptica photonics) was used. The laser beams were focused to the backfocal plane of an oil-immersion objective (100×, NA = 1.4, UPlanSApo, Olympus). The fluorescence signal was spectrally filtered by emission filters (ET 700/75, Chroma or BrightLine 531/40, Semrock) and imaged on an EMCCD camera (Ixon X3/Andor, preGain 5.1, EM-gain 200–400, 30 ms integration time) with a pixel size of 100 nm. An actively stabilizing optical table (TS-300, JRS Scientific Instruments) and nosepiece stage (IX2-NPS, Olympus) were used for a significant reduction of sample and setup drift.

### Data analysis

#### Photophysical properties of single eYFP colocalized with one ATTO647N dye

The fluorescence signal from ATTO647N and eYFP from the same 12HB structures was acquired sequentially (ca. 4000 frames for each fluorophore). The analysis of acquired 2-D measurements was carried out with custom-made software written in LabVIEW 2011 in dual-view configuration^38^. For generation of TIRF images (Fig. 1b) the first 20 frames of the raw video were summed up. Using an automated spot-finding algorithm^31^ molecule spots were selected and their corresponding transients were generated. For discrimination between ON- and OFF-states fluorescence transients were transferred into a binary representation according to the above mentioned spot-finding algorithm.

#### Localization-based microscopy

Each frame of a TIRF video (ca. 8000 per video) was analyzed with an in-house designed software based on MATLAB by applying a spot-finding algorithm with a contrast value of 1.8 and subsequent two-dimensional Gaussian fitting to determine the center of fluorescent spots^31^. For reconstruction of the super-resolution images and distance evaluation, the obtained set of localization coordinates for the whole TIRF video was binned into a 2-dimensional matrix in a modified version of CAEOBS^19^,^28^,(Computer Aided Evaluation of Origami Based Standards) based on LabVIEW 2011. The pixel binning was 10 nm.

Chromatic aberration was corrected by use of multicolor fiducial markers (100 nm diameter, Invitrogen, T7279)^39^.

## Additional Information

**How to cite this article**: Jusuk, I. *et al.* Super-Resolution Imaging Conditions for enhanced Yellow Fluorescent Protein (eYFP) Demonstrated on DNA Origami Nanorulers. *Sci. Rep.* **5**, 14075; doi: 10.1038/srep14075 (2015).

## Supplementary Material

Supplementary Information
