# Supplementary material for: Super-Resolution Imaging Conditions for enhanced Yellow Fluorescent Protein (eYFP) Demonstrated on DNA Origami Nanorulers
Source: Sci Rep. 2015 Sep 16;5:14075. doi: 10.1038/srep14075 (PMC4571581; doi:10.1038/srep14075)
Supplement: Supplementary Information [file srep14075-s1.pdf]

Supplementary Information

Super-Resolution Imaging Conditions for enhanced Yellow Fluorescent Protein (eYFP) Demonstrated on DNA Origami Nanorulers

Ija Jusuk, Carolin Vietz, Mario Raab, Thorben Dammeyer, Philip Tinnefeld*

Institute for Physical & Theoretical Chemistry, and Braunschweig Integrated Centre of Systems Biology (BRICS), and Laboratory for Emerging Nanometrology (LENA), Braunschweig University of Technology, Braunschweig 38106, Germany, *p.tinnefeld@tu-braunschweig.de

1. Experimental methods

Reagents. BSA, BSA-biotin, 2-mercaptoethanol (ME), D-(+) glucose, glucose oxidase, catalase were purchased from Sigma Aldrich and NeutrAvidin from Thermo Fisher Scientific. All modified and unmodified DNA-staple strands were purchased from Eurofins-MWG-Biotech. M13 bacteriophage ssDNA scaffold (8064 nt long, derived from M13mp18 [1]) was produced in our lab. t50-buffer is defined as 50 mM Tris, 50 mM NaCl, 100 mM $MgCl_2$, pH 7.4.

Coding sequence eYFP-His$_6$-Cys:

MAISDPNSIVSKGEELFTGVVPILVELDGDVNGHKFSVSGEGEGDATYGKLTLKFICTTGKLPVPWPTLVTTFGYGLQCFARYP
DHMKQHDFFKSAMPEGYVQERTIFFKDDGNYKTRAEVKFEGDTLVNRIELKGIDFKEDGNILGHKLEYNYNSHNVYIMADK
QKNGIKVNFKIRHNIEDGSVQLADHYQQNTPIGDGPVLLPDNHYLSYQSKLSKDPNEKRDHMVLLEFVTAAGITLGMDELYK
HHHHHHC

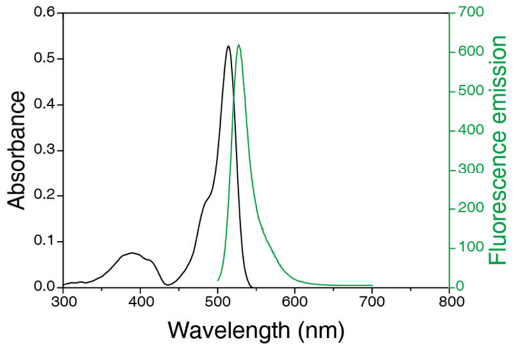

Figure S1. Absorption and fluorescence emission spectra of purified eYFP in t50 (Tris, pH 7.4, ca. 15 µM).

Generation of semisynthetic fluorescent protein DNA–conjugates. The hetero-bispecific crosslinker sulfosuccinimidy 4-[N-maleimidomethyl]-cyclohexane-1-carboxylate (sSMCC) was used to conjugate the protein to aminomodified DNA oligonucleotide as previously suggested.[2] The oligonucleotide (21 bases, 5'-NH_2-C_6H_{12}-GTG ATG TAG GTG GTA GAG GAA) was reacted with sSMCC and the YFP activated with Dithiothreithol (DTT). The activated protein and the maleimide activated oligonucleotide were purified by gel permeation chromatography on a Superdex 200 10/300 global column using the Äkta purifier system (GEhealthcare) and the purified products mixed and reacted at 23 °C for 12 h in the dark. The conjugate was purified by anion-exchange chromatography on a MonoQ HR5/5 column (GEHealthcare) by elution with gradually increasing NaCl concentration from 0 to 0.7 M (Figure S2a). The successful synthesis of protein-DNA conjugates was further confirmed by 15 % native PAGE and 15 % SDS-PAGE analysis. For native PAGE analysis (Figure S2c) the peak (see Figure S2a) of eYFP-DNA was hybridized with a complementary Atto655 labeled oligonucleotide. The colocalization (yellow) of the emission signal of the fluorescent protein (green) and Atto655 dye (red) was revealed using a Typhoon FLA7000 fluorescence scanner (GEhealthcare) (Figure S2c, lane 3). From SDS-PAGE analysis, the molar mass of the eYFP-DNA peak was determined to be ca. 35 kDa which corresponded to the 1:1 conjugation stoichiometry (Figure S2b, lane 2).

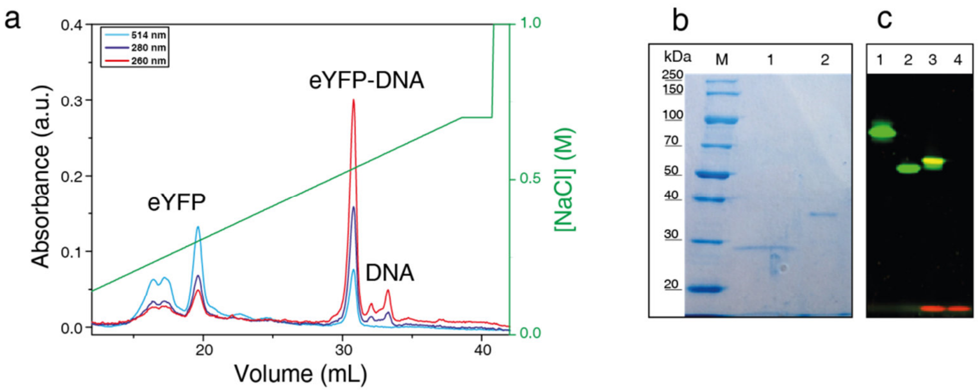

Figure S2. a) Purification of eYFP-DNA conjugate by anion-exchange chromatography monitoring the absorbance at 260 nm (DNA, red), 280 nm (protein, dark blue), 514 nm (eYFP, light blue) and NaCl-gradient (green). b) 15 % SDS-PAGE analysis with lane 1: purified eYFP (28 kDa), lane 2: eYFP-DNA peak from Figure S2a (35 kDa), and lane M: PageRuler Unstained Broad Range Protein Ladder (Thermo Fisher Scientific). c) 15 % native-PAGE analysis with lane 1: purified and activated eYFP, lane 2: eYFP-DNA peak, lane 3: eYFP-DNA peak hybridized with a complementary ATTO655 labeled oligonucleotide, and lane 4: ATTO655 labeled oligonucleotide (21 bases).

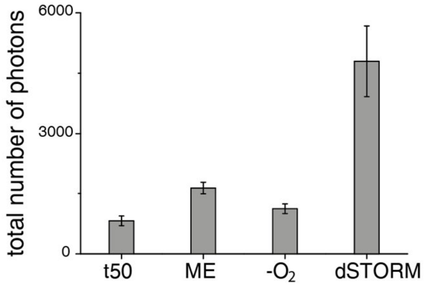

Figure S3. Total number of photons of eYFP-DNA immobilized on 12 helix bundle DNA origami structures under four different buffer conditions.

2. Additional information on super-resolution data

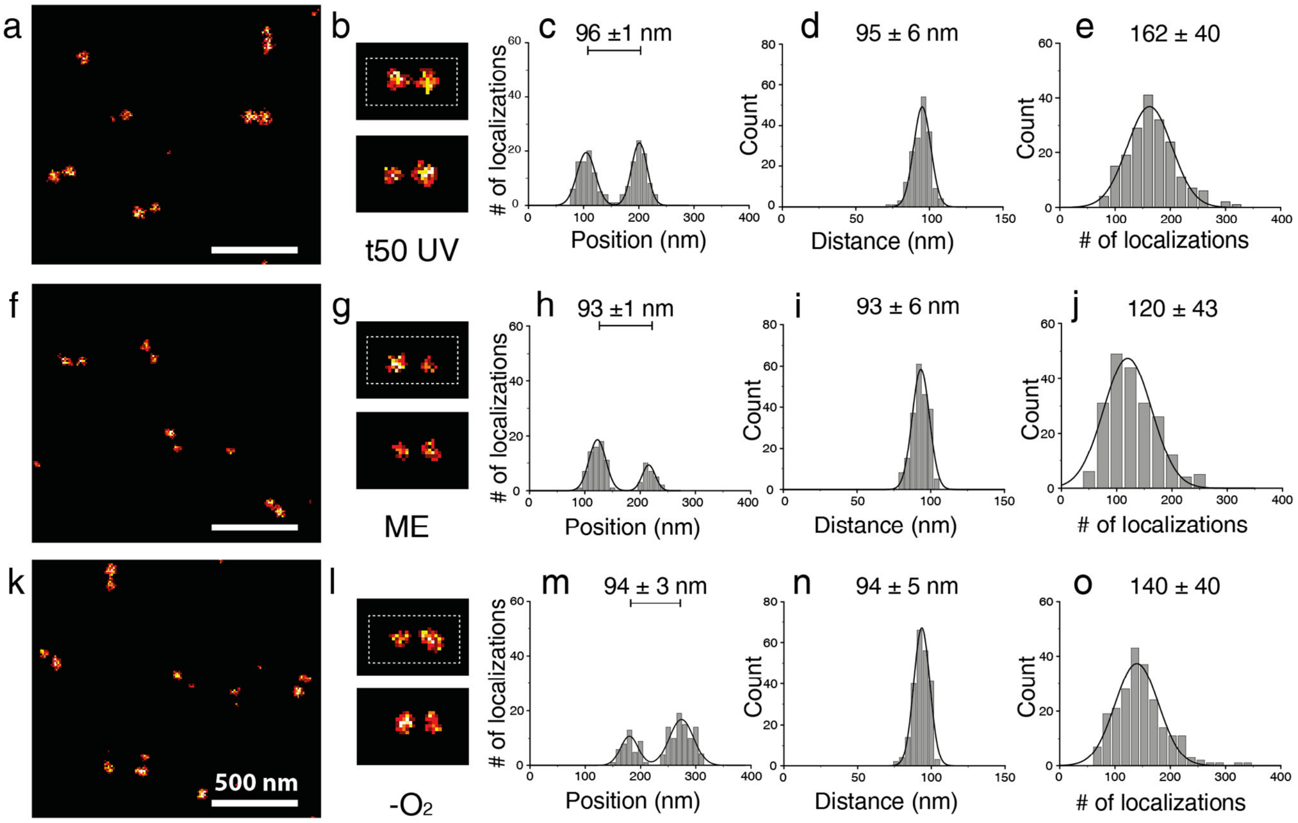

Figure S4. Additional super-resolution data of eYFP labeled 12 helix bundle DNA origamis obtained by localization-based super-resolution microscopy. Super-resolution images of single DNA origami structures with intermark distance of 100 nm a),b) in t50 buffer with UV reactivation, f),g), with 70 mM ME, k),l) under oxygen depletion. For determination of distances, cross-section histograms of double spots were fitted with a sum of two Gaussian functions. Exemplary cross-section histograms of marked double spots c) in t50 buffer with UV reactivation, h) 70mM ME, m) oxygen depletion. Histograms of measured distances were fitted by a Gaussian function as shown: d) t50 buffer with UV reactivation, i) 70 mM ME, n) oxygen depletion. For each evaluated double spot the number of obtained localizations was determined as shown: e) t50 buffer with UV reactivation, j) 70 mM ME, o) oxygen depletion. For statistic evaluation more than 200 structures were analyzed for each distance and buffer condition, errors in (h-j, m-o) are standard deviations of Gaussian fits.

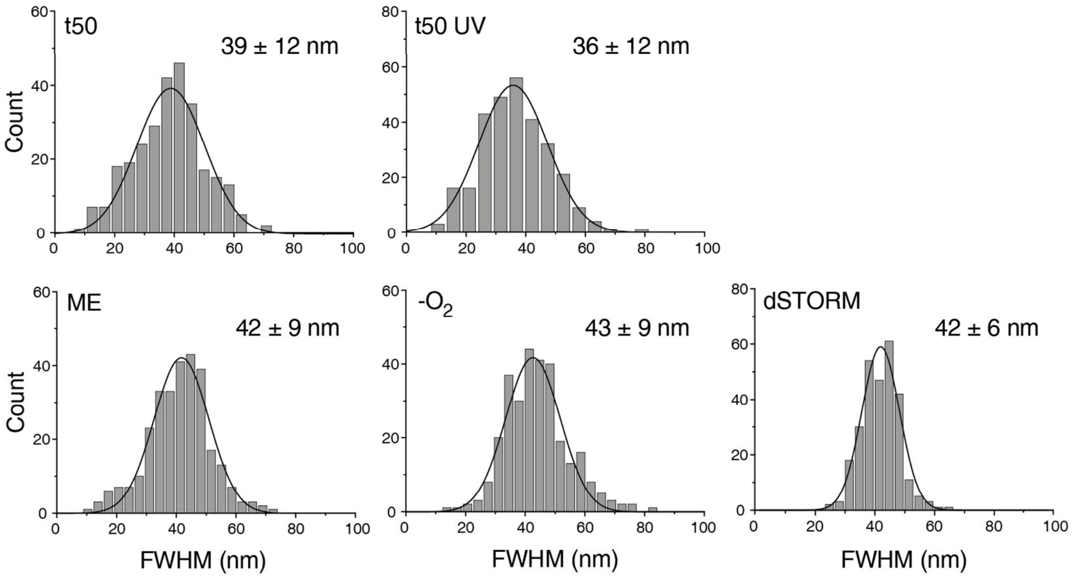

Figure S5. Full width at half maximum-distribution (FWHM) of resolved marks of eYFP modified 12helix bundle DNA origamis with designed distance of 100 nm in t50-buffer, t50-buffer with UV reactivation, 70 mM ME, enzymatic depletion of oxygen and dSTORM-buffer. For statistic evaluation more than 200 structures were analyzed for each distance and buffer condition, errors are standard deviation of Gaussian fits.

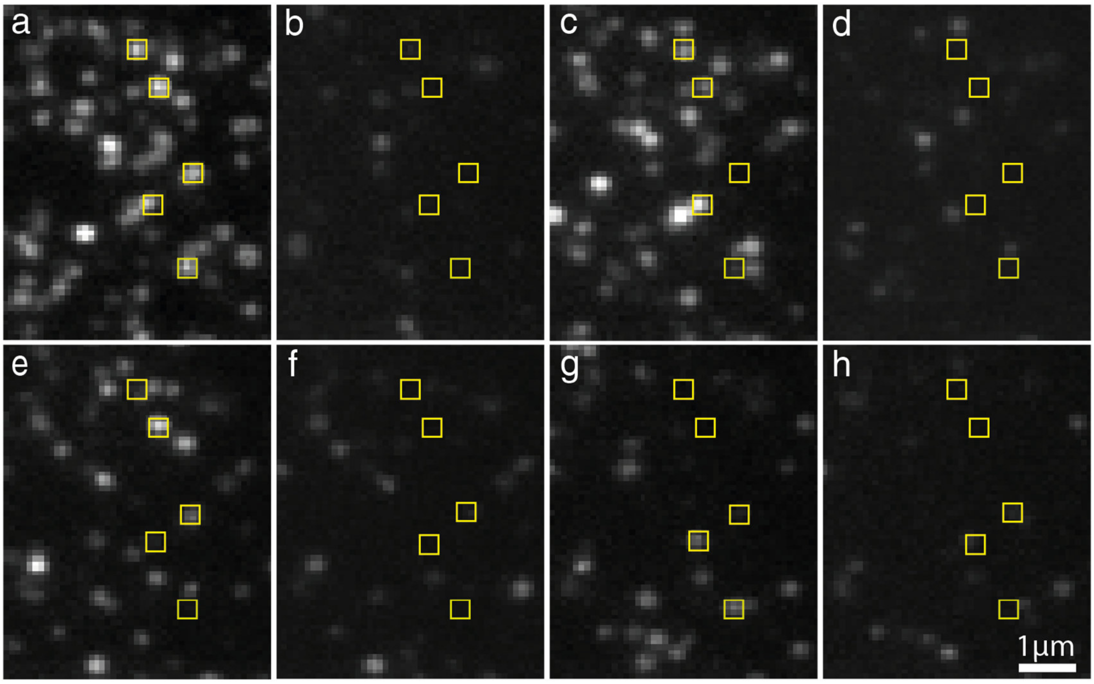

Figure S6. Multiple photoactivation cycles of eYFP modified 12 helix bundle DNA origamis with designed distance of 100 nm by UV (405 nm, at ca. 500 Wcm^{-2}) and subsequent excitation with 488 nm at 800 Wcm^{-2} in t50-buffer. Sum of 60 TIRF-video frames with 488 nm-excitation before (a, b, d, f, h) and after (c, e, g) short 405-nm reactivation pulses of ca. 3 sec. Most of the molecules could be activated maximally up to two times.

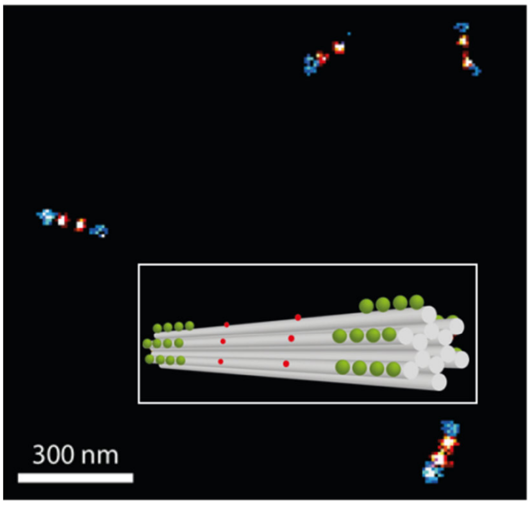

Figure S7. Two color super-resolution image of 12 helix bundle DNA origamis with eYFP-DNA conjugates (outer 36 binding sides) and Alexa647-Poly-(A)22-DNA (interior 20 binding sides) with designed distances of 160 nm and 70 nm, respectively. The fluorescence signal from Alexa647 and eYFP from the same 12HB nanostructures was acquired sequentially after excitation with 644 nm at laser power of ca. 1 $kWcm^{-2}$ and with 488 nm at ca. 800 Wcm^{-2}. Inset sketch of 12HB DNA origami for two color super-resolution imaging with eYFP (green spheres) and Alexa647 dyes (red spheres).

3. DNA sequences

Table S1. Modified DNA staple for 12 HB

Start, End, Sequence 5' - 3'	Modification
4[83],4[61],GTACATCGACATCGTTAACGGCA	5'-TTTTTT-biotin
4[461],4[439],AAGAAACAATGACCGGAAACGTC	5'-TTTTTT-biotin
4[251],4[229],AACGCCAAAAGGCGGATGGCTTA	5'-TTTTTT-biotin
5[665],4[672],ATACCACCATCAGTGAGGCCAAACCGTTGTAGCAA	3'-TTTTTT-biotin
11[399],6[406],GTTTGATGGTGGTTCAGAACCCCGCCTCACAGAAT	5'-TTT-Atto647N

Table S2. Protruding DNA staples for colocalization of eYFP with Atto647N 12 HB (1), 100 nm (2) 160 nm (3), 2-color super-resolution (4); TTTCCTCTACCACCTACATCAC is the complementary part to eYFP-DNA conjugate

Start, End, Sequence 5' - 3'	
8[657],8[635], GTATTAGAGCCGTCAATAGATAAATA**TTTCCTCTACCACCTACATCAC**	1
3[434],3[456],AAAAGGGCGACAATTATTTATCCATATTTCCTCTACCACCTACATCAC	2
10[457],11[470],AAAAGATAGGGTTGAGTGTATATTTCCTCTACCACCTACATCAC	2
6[435],7[440],GCCCCGAGTTTTCATCGGCATATATTTCCTCTACCACCTACATCAC	2
6[461],8[448] ATTATTGTTAGCGATTAAGACTCCTTATATTTCCTCTACCACCTACATCAC	2
2[433],3[433],AGGGACAAAATCTTCCAGCGCCAAAGACATATTTCCTCTACCACCTACATCAC	2
0[431],11[440],ATTCAAGGGGAAGGTAAATGTGGCAAATAAATCATATTTCCTCTACCACCTACATCAC	2
10[427],0[413],AGAACGTAGAGCCTTAAAGGTGAATTAATATTTCCTCTACCACCTACATCAC	2
0[389],11[398],GAATTGTAGCCAGAATGGATCAGAGCAAATCCTATATTTCCTCTACCACCTACATCAC	2

7[422],8[406],AGCGCCACCACGGAATACGCCTCAGACCAGAGCCACCACCATATTTCCTCTACCACCTACATCAC	2
0[412],0[390],TCACCGTCACCGGCGCAGTCTCTATATTTCCTCTACCACCTACATCAC	2
0[454],0[432],AGACGGGAGAATTGACGGAAATTATATTTCCTCTACCACCTACATCAC	2
2[391],3[391],ATTAAAATAAGTGCGACGATTGGCCTTGATATTTCCTCTACCACCTACATCAC	2
10[415],11[428]CCTCCGAAATCGGCAAAATATATTTCCTCTACCACCTACATCAC	2
3[392],3[414],ATATTCACAAACAAATTCATATGATATTTCCTCTACCACCTACATCAC	2
11[471],0[455],TGTTCCAACGCTAACGAACAAGTCAGCAGGGAAGCGCATTATATTTCCTCTACCACCTACATCAC	2
8[489],8[467],AAAACGGAATACCCAAAAGAACTATATTTCCTCTACCACCTACATCAC	2
5[413],4[420],AGAGTTTATACCAGTAGCACCTGAAACCATCGATAATATTTCCTCTACCACCTACATCAC	2
4[438], 6[434]ACCAAATTACCAGGTCATA	2
7[464], 6[460]AAGCACAGAGCCTA	2
11[429], 10[426]CCCTTCATATAAA	2
0[118],0[96],CGAGTAACAACCGTTTACCAGTCATATTTCCTCTACCACCTACATCAC	3,4
0[137],11[146],CATCAGCGTCTGGCCTTCCACAGGAACCTGGGGATATTTCCTCTACCACCTACATCAC	3,4
0[160],0[138],GATAAAAATTTTTAGCCAGCTTTATATTTCCTCTACCACCTACATCAC	3,4
0[95],11[104],CCGGAAGACGTACAGCGCCGCGATTACAATTCCATATTTCCTCTACCACCTACATCAC	3,4
10[121],11[134],GGGCCGGAAGCATAAAGTGATATTTCCTCTACCACCTACATCAC	3,4
10[163],11[176],TCAGCTAACTCACATTAATATATTTCCTCTACCACCTACATCAC	3,4
11[135],0[119],TAAAGGATTGTATAAGCGCACAAACGACATTAAATGTGAGATATTTCCTCTACCACCTACATCAC	3,4
11[177],0[161],TGCGTACTAATAGTAGTTGAAATGCATATTTCAACGCAAGATATTTCCTCTACCACCTACATCAC	3,4
2[139],3[139],TTCGCGGATTGATTGCTCATTTTTTAACATATTTCCTCTACCACCTACATCAC	3,4
2[97],3[97],GCGAAAGACGCAAAGCCGCCACGGGAACATATTTCCTCTACCACCTACATCAC	3,4
3[140],3[162],CAATAGGAACGCAAATTAAGCAAATATTTCCTCTACCACCTACATCAC	3,4
3[98],3[120],GGATAACCTCACAATTTTTGTTAATATTTCCTCTACCACCTACATCAC	3,4
4[144],7[146],CGTAAAGGTCACGAAACCAGGCAATAGCACCGCTTCTGGTATATTTCCTCTACCACCTACATCAC	3,4
7[105],7[127]GAAAGATCGCACTCCAGCCAGCTATATTTCCTCTACCACCTACATCAC	3,4
7[128],8[112],TTCCGAATTGTAAACGTGTCGCCAGCATCGGTGCGGGCCTATATTTCCTCTACCACCTACATCAC	3,4
7[147],7[169],GCTAATGCCGGAGAGGGTAGCTAATATTTCCTCTACCACCTACATCAC	3,4
7[170],8[154],TTTTTATCCAATAAATCTCTACCCCGGTAAAACTAGCATGATATTTCCTCTACCACCTACATCAC	3,4
8[153],8[131]TCAGGCTGCGCAACTGTTGGGAAATATTTCCTCTACCACCTACATCAC	3,4
8[195],8[173],TTAACAAGAGAATCGATGAACGGATATTTCCTCTACCACCTACATCAC	3,4
0[599],11[608],TTCTGGAATAATCCTGATTTTGCCCGGCCGTAAATATTTCCTCTACCACCTACATCAC	2,3,4
0[622],0[600],AAGATAAAACAGTTGGATTATACATATTTCCTCTACCACCTACATCAC	2,3,4
0[641],11[650],CCGAACCCCCTAAAACATCGACCAGTTTAGAGCATATTTCCTCTACCACCTACATCAC	2,3,4
0[664],0[642],GATTTTAGACAGGCATTAAAAATAATATTTCCTCTACCACCTACATCAC	2,3,4
10[583],11[596],TGGCAAGTTTTTTGGGGTCATATTTCCTCTACCACCTACATCAC	2,3,4
10[625],11[638],AACACCCTAAAGGGAGCCCATATTTCCTCTACCACCTACATCAC	2,3,4
11[639],0[623],CCGATAATAAAAGGGACTTAACACCGCGAACCACCAGCAGATATTTCCTCTACCACCTACATCAC	2,3,4
2[601],3[601],TCAATAATAAAGTGTATCATCATATTCCATATTTCCTCTACCACCTACATCAC	2,3,4
2[643],3[643],GATAGTGCAACATGATATTTTTGAATGGATATTTCCTCTACCACCTACATCAC	2,3,4
3[602],3[624],TGATTATCAGATATACGTGGCACATATTTCCTCTACCACCTACATCAC	2,3,4
3[644],3[666],CTATTAGTCTTTCGCCGCTACAGATATTTCCTCTACCACCTACATCAC	2,3,4
4[648],7[650],GCATCGAGCCAGATATCTTTAGGACCTGAGGAAGGTTATCATATTTCCTCTACCACCTACATCAC	2,3,4
7[609],7[631],ATTTGGCAAATCAACAGTTGAAAATATTTCCTCTACCACCTACATCAC	2,3,4
7[632],8[616],GGAATAACAGAGATAGACATACAAACTTGAGGATTTAGAAATATTTCCTCTACCACCTACATCAC	2,3,4

7[651],7[673],TAAGTAGAAGAACTCAAACTATCGATATTTCCTCTACCACCTACATCAC	2,3,4
7[674],8[658],GCCTTACGCTGCGCGTAAAATTATTTTTTGACGCTCAATCATATTTCCTCTACCACCTACATCAC	2,3,4
8[615],8[593],GTTGAAACAAACATCAAGAAAACATATTTCCTCTACCACCTACATCAC	2,3,4
8[657],8[635],GTATTAGAGCCGTCAATAGATAAATATTTCCTCTACCACCTACATCAC	2,3,4
7[441],7[463],TTGAAGCCCTTTTTAAGAAAAGTATATTTTTTTTTTTTTTTTTTTTTTTTT	4
0[496],0[474],TCCCATCCTAATGAGAATAACATATA(T)25	4
3[476],3[498],TTTTTTGTTTAATAAAGTAATTCATA(T)25	4
7[506],8[490],AAATCAGCCAGTAATAACACTATTTTTGAAGCCTTAAATCATA(T)25	4
4[480],7[482],TAAGCCAGAGAGCCAGAAGGAAACTCGATAGCCGAACAAAATA(T)25	4
10[457],11[470],AAAAGATAGGGTTGAGTGTATA(T)25	4
8[489],8[467],AAAACGGAATACCCAAAAGAACTATA(T)25	4
2[475],3[475],AAATAGGTAATTTACAAATAAGAAACGAATA(T)25	4
11[513],0[497],CTCCAATTTAGGCAGAGACAATCAATCAAGAAAAATAATAATA(T)25	4
0[473],11[482],AAAAAAGGCAGCCTTTACAATCTTACCAGTTTGATA(T)25	4
0[263],11[272],CAGTCTTGATTTTAAGAACTCAACGTTGCGTATATA(T)25	4
3[266],3[288],AACTTTAATCATGGGTAGCAACGATA(T)25	4
7[231],7[253],TGCAACACTATCATAACCCTCGTATA(T)25	4
0[286],0[264],AAAACGAAAGAGGCTCATTATACATA(T)25	4
2[265],3[265],TATGCATTACAGAGGATGGTTTAATTTCATA(T)25	4
7[296],8[280],AGACGTCGTCACCCTCAGATCTTGACGCTGGCTGACCTTCATA(T)25	4
11[303],0[287],GTGAGTTAAAGGCCGCTGACACTCATGAAGGCACCAACCTATA(T)25	4
8[279],8[257],ATTTTGCCAGAGGGGGTAATAGTATA(T)25	4
4[270],7[272],TCAACATCAGTTAAATAGCGAGAGTGAGACGACGATAAAAATA(T)25	4
10[247],11[260],AATAACGCGCGGGGAGAGGATA(T)25	4

Table S3. 12HB-unmodified DNA-staples. Colocalization of eYFP with Atto647N (1), super-resolution with 100 nm (2), super-resolution with 160 nm (3), 2-color super-resolution with YFP and Alexa647 (4)

**Start, End, Sequence, 5' - 3'**	
0[389],11[398],GAATTGTAGCCAGAATGGATCAGAGCAAATCCT	1,3,4
0[118],0[96],CGAGTAACAACCGTTTACCAGTC	1,2
0[137],11[146],CATCAGCGTCTGGCCTTCCACAGGAACCTGGGG	1,2
0[160],0[138],GATAAAAATTTTTAGCCAGCTTT	1,2
0[179],11[188],GCCTTATACCCTGTAATACCAATTCTTGCGCTC	1,2,3,4
0[202],0[180],GACCGGAAGCAATTGCGGGAGAA	1,2,3,4
0[221],11[230],CGAGCACAGACTTCAAATACCTCAAAAGCTGCA	1,2,3,4
0[244],0[222],AAAAATCTACGTGCGTTTTAATT	1,2,3,4
0[263],11[272],CAGTCTTGATTTTAAGAACTCAACGTTGCGTAT	1,2,3
0[286],0[264],AAAACGAAAGAGGCTCATTATAC	1,2,3
0[305],11[314],ACTACCTTTAAACGGGTAACAGGGAGACGGGCA	1,2,3,4
0[328],0[306],TTGTCGTCTTTCTACGTAATGCC	1,2,3,4
0[347],11[363],AGCGTATCATTCCACAGACCCGCCACAGTTGCAGCAAGCG	1,2,3,4
0[370],0[348],GCGTCATACATGCCCTCATAGTT	1,2,3,4
0[412],0[390],TCACCGTCACCGGCGCAGTCTCT	1,3,4
0[431],11[440],ATTCAAGGGGAAGGTAAATGTGGCAAATAAATC	1,3,4
0[454],0[432],AGACGGGAGAATTGACGGAAATT	1,3,4

0[473],11[482],AAAAAAGGCAGCCTTTACAATCTTACCAGTTTG	1,2,3
0[496],0[474],TCCCATCCTAATGAGAATAACAT	1,2,3
0[515],11[524],CTGAAAACCTGTTTATCAAACATGTAACGTCAA	1,2,3,4
0[53],11[62],CGGTAGTACTCAATCCGCTGCTGGTCATGGTC	1,2,3,4
0[538],0[516],TTAGGTTGGGTTATAGATAAGTC	1,2,3,4
0[557],11[566],TACCTAATATCAAAATCATTCAATATTACGTGA	1,2,3,4
0[580],0[558],TAGAACCTACCAGTCTGAGAGAC	1,2,3,4
0[599],11[608],TTCTGGAATAATCCTGATTTTGCCCGGCCGTAA	1
0[622],0[600],AAGATAAAACAGTTGGATTATAC	1
0[641],11[650],CCGAACCCCCTAAAACATCGACCAGTTTAGAGC	1
0[664],0[642],GATTTTAGACAGGCATTAAAAATA	1
0[76],0[54],GACTTTCTCCGTGGCGCGGTTG	1,2,3,4
0[95],11[104],CCGGAAGACGTACAGCGCCGCGATTACAATTCC	1,2
10[121],11[134],GGGCCGGAAGCATAAAGTG	1,2
10[163],11[176],TCAGCTAACTCACATTAAT	1,2
10[205],11[218],AGCAGTCGGGAAACCTGTC	1,2,3,4
10[247],11[260],AATAACGCGCGGGGAGAGG	1,2,3
10[289],11[302],GATGTTTTTCTTTTCACCA	1,2,3,4
10[331],11[344],TCGTTCACCGCCTGGCCCT	1,2,3,4
10[373],11[386],TACCTGGTTTGCCCCAGCA	1,2,3,4
10[415],11[428],CCTCCGAAATCGGCAAAAT	1,3,4
10[457],11[470],AAAAGATAGGGTTGAGTGT	1,3
10[499],11[512],CTATATTAAAGAACGTGGA	1,2,3,4
10[541],11[554],CATTCTATCAGGGCGATGG	1,2,3,4
10[583],11[596],TGGCAAGTTTTTTGGGGTC	1
10[625],11[638],AACACCCTAAAGGGAGCCC	1
10[667],11[680],AGACGGCGAACGTGGCGAG	1,2,3,4
10[79],11[92],GTATGTGAAATTGTTATCC	1,2,3,4
11[105],6[112],ACACAACATACGAGGGATGTGGCTATTAATCGGCC	1,2,3,4
11[135],0[119],TAAAGGATTGTATAAGCGCACAAACGACATTAAATGTGAG	1,2
11[147],6[154],TGCCTAATGAGTGAGAAAAGCTCATATGTAGCTGA	1,2,3,4
11[177],0[161],TGCGTACTAATAGTAGTTGAAATGCATATTTCAACGCAAG	1,2
11[189],6[196],ACTGCCCGCTTTCCTGAAAAGCTATATTTTAAATA	1,2,3,4
11[219],0[203],GTGCCTGCTTTAAACAGGGAGAGAGTTTCAAAGCGAACCA	1,2,3,4
11[231],6[238],TTAATGAATCGGCCATTCATTCCAATACGCATAGT	1,2,3,4
11[261],0[245],CGGTTAACAAAGCTGCTGTAACAACAAGGACGTTGGGAAG	1,2,3,4
11[273],6[280],TGGGCGCCAGGGTGATTCATTAGAGTAACCTGCTC	1,2,3,4
11[303],0[287],GTGAGTTAAAGGCCGCTGACACTCATGAAGGCACCAACCT	1,2,3
11[315],6[322],ACAGCTGATTGCCCGTCGCTGCGCCCACACGTTGA	1,2,3,4
11[345],0[329],GAGAGCCTCAGAACCGCATTTTCTGTAACGATCTAAAGTT	1,2,3,4
11[364],6[364],GTCCACGCGCCACCTCACCGTTGAAACA	1,2,3,4
11[387],0[371],GGCGACACCACCCTCAGGTTGTACTGTACCGTTCCAGTAA	1,2,3
11[399],6[406],GTTTGATGGTGGTTCAGAACCCCGCCTCACAGAAT	2,3,4
11[429],0[413],CCCTTCATATAAAGAACGTAGAGCCTTAAAGGTGAATTA	1,3,4
11[441],6[448],AAAAGAATAGCCCGATACATACGCAGTAAGCTATC	1,2,3,4

11[471],0[455],TGTTCCAACGCTAACGAACAAGTCAGCAGGGAAGCGCATT	1,3,4
11[483],6[490],GAACAAGAGTCCACCAATTTTTTAGTTGTCGTAGG	1,2,3,4
11[513],0[497],CTCCAATTTAGGCAGAGACAATCAATCAAGAAAAATAATA	1,2,3
11[525],6[532],AGGGCGAAAAACCGATTTAACGTAGGGCAAATACC	1,2,3,4
11[555],0[539],CCCACATGTGAGTGAATAACTGATGCTTTTAACCTCCGGC	1,2,3,4
11[567],6[574],ACCATCACCCAAATAAACAGTTCATTTGATTCGCC	1,2,3,4
11[597],0[581],GAGGTAACGTTATTAATTTTAAAACAAATAATGGAAGGGT	1,2,3,4
11[609],6[616],AGCACTAAATCGGATCGTATTTAGACTTATATCTG	1,2,3,4
11[63],6[70],ATAGCTGTTTCCTGGAACGTCCATAACGCCGTAAA	1,2,3,4
11[639],0[623],CCGATAATAAAAGGGACTTAACACCGCGAACCACCAGCAG	1
11[651],6[658],TTGACGGGGAAAGCTTCACCAGAAATGGCATCACT	1,2,3,4
11[681],10[668],AAAGGGCGCTGGCAAGTATTGGC	1,2,3,4
11[93],0[77],GCTCAAGTTGGGTAACGGGCGGAAAAATTTGTGAGAGATA	1,2,3,4
2[139],3[139],TTCGCGGATTGATTGCTCATTTTTTAAC	1,2
2[181],3[181],TTATGGCCTGAGCACCTCAGAGCATAAA	1,2,3,4
2[223],3[223],CCGAACTTTAATAAAAGCAAAGCGGATT	1,2,3,4
2[265],3[265],TATGCATTACAGAGGATGGTTTAATTTC	1,2,3
2[307],3[307],TTCCATTGACCCAAAGAGGCTTTGAGGA	1,2,3,4
2[349],3[349],TGTAGGGGATTTAGTAACACTGAGTTTC	1,2,3,4
2[391],3[391],ATTAAAATAAGTGCGACGATTGGCCTTG	1,3,4
2[433],3[433],AGGGACAAAATCTTCCAGCGCCAAAGAC	1,3,4
2[475],3[475],AAATAGGTAATTTACAAATAAGAAACGA	1,2,3
2[517],3[517],ACGCGTCGGCTGTAAGACGACGACAATA	1,2,3,4
2[55],3[55],TTCGCCATAAACTCTGGAGGTGTCCAGC	1,2,3,4
2[559],3[559],GAATTATCCAATAACGATAGCTTAGATT	1,2,3,4
2[601],3[601],TCAATAATAAAGTGTATCATCATATTCC	1
2[643],3[643],GATAGTGCAACATGATATTTTTGAATGG	1
2[97],3[97],GCGAAAGACGCAAAGCCGCCACGGGAAC	1,2
3[121],10[122],AATCAGTTAAAACGTGGGAGAAA	1,2,3,4
3[140],3[162],CAATAGGAACGCAAATTAAGCAA	1,2
3[163],10[164],TAAAGAGGCAAAATATTTTATAA	1,2,3,4
3[182],3[204],GCTAAATCGGTTTGACTATTATA	1,2,3,4
3[205],10[206],GTCAGAATCAGGCAGGATTCGCG	1,2,3,4
3[224],3[246],GCATCAAAAAGAAGTAAATTGGG	1,2,3,4
3[247],10[248],CTTGAAAACACCCTAACGGCATA	1,2,3,4
3[266],3[288],AACTTTAATCATGGGTAGCAACG	1,2,3
3[289],10[290],GCTACGACAGCAACTAAAAACCG	1,2,3,4
3[308],3[330],CTAAAGACTTTTAGGAACCCATG	1,2,3,4
3[331],10[332],TACCGGGATAGCAATGAATATAT	1,2,3,4
3[350],3[372],GTCACCAGTACAAGGTTGAGGCA	1,2,3,4
3[373],10[374],GGTCACGCCAGCACAGGAGTTAG	1,2,3,4
3[392],3[414],ATATTCACAAACAAATTCATATG	1,3,4
3[415],10[416],GTTTATGTCACATGGGAATCCAC	1,2,3
3[434],3[456],AAAAGGGCGACAATTATTTATCC	1,3,4
3[457],10[458],CAATCCAAAATACTGAACAGTAG	1,2,3,4

3[476],3[498],TTTTTTGTTTAATAAAGTAATTC	1,2,3
3[499],10[500],TGTCCAAGTACCAGAAACCCCAG	1,2,3,4
3[518],3[540],AACAACATGTTCATCCTTGAAAA	1,2,3,4
3[541],10[542],CATAGTTAATTTGTAAATGTCGC	1,2,3,4
3[56],3[78],ATCAGCGGGGTCAGCTTTCAGAG	1,2,3,4
3[560],3[582],AAGACGCTGAGACCAGAAGGAGC	1,2,3,4
3[583],10[584],GGAATCGGAACATTGCACGTTAA	1,2,3,4
3[602],3[624],TGATTATCAGATATACGTGGCAC	1
3[625],10[626],AGACAACCTGAACAGTATTCGAC	1,2,3,4
3[644],3[666],CTATTAGTCTTTCGCCGCTACAG	1
3[667],0[665],GGCGCCCCGCCGAATCCTGAGAAGTGAGGCCGATTAAAGG	1,2,3,4
3[79],10[80],GTGGAACGACGGGCTCTCAACTT	1,2,3,4
3[98],3[120],GGATAACCTCACAATTTTTGTTA	1,2
4[102],7[104],CCAGCCAAACTTCTGATTGCCGTTTTGGGTAAAGTTAAAC	1,2,3,4
4[125],4[103],GTTTGAGGGGACCTCATTTGCCG	1,2,3,4
4[144],7[146],CGTAAAGGTCACGAAACCAGGCAATAGCACCGCTTCTGGT	1,2
4[167],4[145],CAATATGATATTGATGGGCGCAT	1,2,3,4
4[186],7[188],GAGACAAAGATTATCAGGTCATTGACGAGAGATCTACAAA	1,2,3,4
4[209],4[187],AATGCTGTAGCTGAGAAAGGCCG	1,2,3,4
4[228],7[230],GAGCTTAAGAGGTCCCAATTCTGCAATTCCATATAACAGT	1,2,3,4
4[270],7[272],TCAACATCAGTTAAATAGCGAGAGTGAGACGACGATAAAA	1,2,3
4[293],4[271],AAATTGTGTCGAGAATACCACAT	1,2,3,4
4[312],7[314],ATTTGCCAAGCGGAACTGACCAACGAGTCAATCATAAGGG	1,2,3,4
4[335],4[313],ATTGCGAATAATGTACAACGGAG	1,2,3,4
4[354],7[356],GAAAGTTCAACAATCAGCTTGCTTAGCTTTAATTGTATCG	1,2,3,4
4[377],4[355],CTATTTCGGAACGAGTGAGAATA	1,2,3,4
4[396],7[398],AACAGAGTGCCTGGGGTTTTGCTCACAGAAGGATTAGGAT	1,2,3,4
4[419],4[397],GCAGCACCGTAAGTGCCCGTATA	1,2,3,4
4[438],7[440],ACCAAATTACCAGGTCATAGCCCCGAGTTTTCATCGGCAT	1,3,4
4[480],7[482],TAAGCCAGAGAGCCAGAAGGAAACTCGATAGCCGAACAAA	1,2,3
4[503],4[481],AGCAAGCCGTTTAAGAATTGAGT	1,2,3,4
4[522],7[524],ACCGCATTCCAACGGTATTCTAAGCGAGATATAGAAGGCT	1,2,3,4
4[545],4[523],TGACCTAAATTTTTAAACCAAGT	1,2,3,4
4[564],7[566],TTTAGAACGCGAATTACTAGAAAACTATAAACACCGGAAT	1,2,3,4
4[587],4[565],CATCGGGAGAAATTCAAATATAT	1,2,3,4
4[60],7[62],TCAGAGGTGTGTCGGCCAGAATGAGTGCACTCTGTGGT	1,2,3,4
4[606],7[608],ACAGTTTTTCAGATTTCAATTACCGTCGCAGAGGCGAATT	1,2,3,4
4[629],4[607],CAAATATCAAACCAGATGAATAT	1,2,3,4
4[648],7[650],GCATCGAGCCAGATATCTTTAGGACCTGAGGAAGGTTATC	1
4[671],4[649],TACTTCTTTGATAAAAATCTAAA	1,2,3,4
5[119],4[126],CATAATATTCCGTAATGGGATCCGTGCATCTGCCA	1,2,3,4
5[161],4[168],GTATACAGGTAATGTGTAGGTAGTCAAATCACCAT	1,2,3,4
5[203],4[210],TGTAAATCATGCTCCTTTTGATAATTGCTGAATAT	1,2,3,4
5[245],4[252],CGCCTGACGGTAGAAAGATTCTAATGCAGATACAT	1,2,3,4
5[287],4[294],GCGCAGCGACCAGCGATTATATATCATCGCCTGAT	1,2,3,4

5[329],4[336],TTCATTTTCTGCTAAACAACTGAACAACTAAAGGA	1,2,3,4
5[371],4[378],ATCAGAGCCTTTAACGGGGTCTTAATGCCCCCTGC	1,2,3,4
5[413],4[420],AGAGTTTATACCAGTAGCACCTGAAACCATCGATA	1,2,3,4
5[455],4[462],CATGCCAGTGAGCGCTAATATCCAATAATAAGAGC	1,2,3,4
5[497],4[504],TTGAGAATATCTTTCCTTATCACTCATCGAGAACA	1,2,3,4
5[539],4[546],TTCGCTATTCGCAAGACAAAGTTAATTTCATCTTC	1,2,3,4
5[581],4[588],ACATCATTTAAATTGCGTAGAAACAGTACCTTTTA	1,2,3,4
5[623],4[630],ATACCCTTCGTGCCACGCTGAACCTTGCTGAACCT	1,2,3,4
5[77],4[84],AACGTTGTAGAAACAGCGGATAGTTGGGCGGTTGT	1,2,3,4
6[111],2[98],TCAGGTGAAATTTCTACGGAAACAATCG	1,2,3,4
6[153],2[140],TAAATCGGTTGGTGCACATCAAAAATAA	1,2,3,4
6[195],2[182],TGCAACTCAAAAGGCCGTACCAAAAACA	1,2,3,4
6[237],2[224],AAGAGATTCATTTTGTTTAAGAGGAAGC	1,2,3,4
6[279],2[266],CATGTCAGAGATTTGATGTGAATTACCT	1,2,3,4
6[321],2[308],AAATCCCCGAAACAATTCATGAGGAAGT	1,2,3,4
6[363],2[350],TGAAATTGTTTCAGGGAACTACAACGCC	1,2,3,4
6[405],2[392],CAAGTGCTGAGTAAGAAAATAAATCCTC	1,2,3,4
6[447],2[434],TTACCTCTTAGCAAATTTCAACCGATTG	1,2,3,4
6[489],2[476],AATCATAATAACCCGGCGTCAAAAATGA	1,2,3,4
6[531],2[518],GACCGTCGAACGGGGAAGCTAATGCAGA	1,2,3,4
6[573],2[560],TGATTTAGAAAACTCAAGAGTCAATAGT	1,2,3,4
6[615],2[602],GTCAGTCGTTTAACGAGATGGCAATTCA	1,2,3,4
6[657],2[644],TGCCTGAACAGCAAATGAATGCGCGAACT	1,2,3,4
6[69],2[56],AAAAGTGTCAGCAACAATTGCAGGCGCT	1,2,3,4
7[105],7[127],GAAAGATCGCACTCCAGCCAGCT	1,2
7[128],8[112],TTCCGAATTGTAAACGTGTCGCCAGCATCGGTGCGGGCCT	1,2
7[147],7[169],GCTAATGCCGGAGAGGGTAGCTA	1,2
7[170],8[154],TTTTTATCCAATAAATCTCTACCCCGGTAAAACTAGCATG	1,2
7[189],7[211],GGCTAAAGTACGGTGTCTGGAAG	1,2,3,4
7[212],8[196],TTTCACGAGAATGACCATTTTCATTTGGTCAATAACCTGT	1,2,3,4
7[231],7[253],TGCAACACTATCATAACCCTCGT	1,2,3
7[254],8[238],TTACCAATAAGGCTTGCAGTGCGGAAGTTTAGACTGGATA	1,2,3,4
7[273],7[295],ACTACTTAGCCGGAACGAGGCGC	1,2,3,4
7[296],8[280],AGACGTCGTCACCCTCAGATCTTGACGCTGGCTGACCTTC	1,2,3
7[315],7[337],AATCCAAAAAAAAGGCTCCAAAA	1,2,3,4
7[338],8[322],GGAGCAGCCACCACCCTTCGCATAACGACAATGACAACAA	1,2,3,4
7[357],7[379],GTGTATTAAGAGGCTGAGACTCC	1,2,3,4
7[380],8[364],TCAAGCAGAACCACCACTCACTCAGGTAGCCCGGAATAGG	1,2,3,4
7[399],7[421],TATTGCCTTTAGCGTCAGACTGT	1,2,3,4
7[422],8[406],AGCGCCACCACGGAATACGCCTCAGACCAGAGCCACCACC	1,3,4
7[441],7[463],TTGAAGCCCTTTTTAAGAAAAGT	1,2,3
7[464],8[448],AAGCACAGAGCCTAATTATTGTTAGCGATTAAGACTCCTT	1,3,4
7[483],7[505],GTTTACCGCGCCCAATAGCAAGC	1,2,3,4
7[506],8[490],AAATCAGCCAGTAATAACACTATTTTTGAAGCCTTAAATC	1,2,3
7[525],7[547],TATGTGATAAATAAGGCGTTAAA	1,2,3,4

7[548],8[532],TAAGATCTGTAAATCGTTGTTAATTGTAAAGCCAACGCTC	1,2,3,4
7[567],7[589],CAGCTTTGAATACCAAGTTACAA	1,2,3,4
7[590],8[574],AATCGTTGAGTAACATTGGAATTACCTAATTACATTTAAC	1,2,3,4
7[609],7[631],ATTTGGCAAATCAACAGTTGAAA	1
7[63],7[85],GCCCGCACAGGCGGCCTTTAGTG	1,2,3,4
7[632],8[616],GGAATAACAGAGATAGACATACAAACTTGAGGATTTAGAA	1
7[651],7[673],TAAGTAGAAGAACTCAAACTATCG	1
7[674],8[658],GCCTTACGCTGCGCGTAAAATTATTTTTGACGCTCAATC	1
7[86],8[70],ATGAATCCCAGTCACGATCGAACGTGCCGGCCAGAGCACA	1,2,3,4
8[111],8[89],CTTTTTTTCGTCTCGTCGCTGGC	1,2,3,4
8[130],9[146],GGGCGTGAAATATTAGCGCCATTCGC	1,2,3,4
8[153],8[131],TCAGGCTGCGCAACTGTTGGGAA	1,2
8[172],9[188],TAATCGTAGCATTACCTGAGAGTCTG	1,2,3,4
8[195],8[173],TTAACAAGAGAATCGATGAACGG	1,2
8[214],9[230],CAAATGGTTCAGAAGAACGAGTAGAT	1,2,3,4
8[237],8[215],GCTTGACCATTAGATACATTTCG	1,2,3,4
8[256],9[272],AAAATTCCATTCAGGCTTTTGCAAAA	1,2,3,4
8[279],8[257],ATTTTGCCAGAGGGGGTAATAGT	1,2,3
8[298],9[314],CATAGAATTTGCGGTTTGAAAGAGGA	1,2,3,4
8[321],8[299],CCGAACGGTGTACAGACCAGGCG	1,2,3,4
8[340],9[356],GCGCCCGCACCCTCTCGAGGTGAATT	1,2,3,4
8[363],8[341],TGAACAGCTTGATACCGATAGTT	1,2,3,4
8[382],9[398],AAGTAAGAGCCGCCAGTACCAGGCGG	1,2,3,4
8[405],8[383],GGTGCCGTCGAGAGGGTTGATAT	1,2,3,4
8[424],9[440],CGGAAGCACGCAAACTTATTAGCGTT	1,2,3,4
8[447],8[425],ATTCTTTTCATAATCAAAATCAC	1,2,3,4
8[46],9[62],CAGCATCAACCGCACGGCGGGCCGTT	1,2,3,4
8[466],9[482],GGCATAAGCGTCTTCGAGGAAACGCA	1,2,3,4
8[489],8[467],AAAACGGAATACCCAAAAGAACT	1,3
8[508],9[524],GGTTTGCGCATTTTAACGCGAGGCGT	1,2,3,4
8[531],8[509],AACGAACCTCCCGACTTGCGGGA	1,2,3,4
8[550],9[566],CAGTAAGAACCTTGAGCCTGTTTAGT	1,2,3
8[573],8[551],AAATGCGTTATACAAATTCTTAC	1,2,3,4
8[592],9[608],AAAATTTTTTAAAATGAGCAAAAGAA	1,2,3,4
8[615],8[593],GTTGAAACAAACATCAAGAAAAC	1
8[634],9[650],TACATAAATTCTGGGCACTAACAACT	1,2,3,4
8[69],8[47],TCGGTCATACCGGGGGTTTCTGC	1,2,3,4
8[88],9[104],AGCCTCCCCAGGGTCCGGCAAACGCG	1,2,3,4
9[105],5[118],GTCCGTCCTGCAAGATCGTCGGATTCTCTTCGCATTGGACGA	1,2,3,4
9[147],5[160],CATTCAACCCAAAATGTAGAACCCTCATGAATTAGTACAACC	1,2,3,4
9[189],5[202],GAGCAAGGTGGCATTTACTCCAACAGGTTCTTTACGTCAACA	1,2,3,4
9[231],5[244],TTAGTGTGAATCCCTCTAATAAAACGAAAGAACGATGAATTA	1,2,3,4
9[273],5[286],GAAGTCAACCCAAATGGCAAAAGAATACTCGGAACAGAATCC	1,2,3,4
9[315],5[328],CAGATATAGGCTTGAACAGACGTTAGTAAAGCCCAAAAATTT	1,2,3,4
9[357],5[370],TCTTATACTCAGAAAGGCTTTTGATGATATTGACACGCTATT	1,2,3,4

9[399],5[412],ATAAGAAGCCACCCAAACTTGAGCCATTATCAATACATCAGT	1,2,3,4
9[441],5[454],TGCCATACATAAAGATTAACTGAACACCAACAGCCGGAATAG	1,2,3,4
9[483],5[496],ATAATGAATCCTGAGATTACGAGCATGTGACAAAAACTTATT	1,2,3,4
9[525],5[538],TTTAGCAAACGCCACAATATAACTATATTCCCTTATAAATGG	1,2,3,4
9[567],5[580],ATCATTTACATAAAAGTATCAAAATTATAAGAAACTTCAATA	1,2,3,4
9[609],5[622],GATGAATAAATCCTGTAGGTGAGGCGGTAGCGTAAGTCCTCA	1,2,3,4
9[63],5[76],TTCACCTAGCGTGGCGGGTGAAGGGATACCAGTGCATAAAAA	1,2,3,4
9[651],5[664],AATAGCTGTCACACGCAACGGTACGCCAGCGCTTAATGTAGTA	1,2,3,4

References

1. Douglas, S.M. et al. Rapid prototyping of 3D DNA-origami shapes with caDNAno. Nucleic Acids Res 37, 5001-5006 (2009).
2. Kukolka, F. & Niemeyer, C.M. Synthesis of fluorescent oligonucleotide-EYFP conjugate: Towards supramolecular construction of semisynthetic biomolecular antennae. Organic & Biomolecular Chemistry 2, 2203-2206 (2004).
